# Parsonage-Turner Syndrome of Unclear Causation: A Case Report

**DOI:** 10.7759/cureus.27244

**Published:** 2022-07-25

**Authors:** Robert E Carrier, Michael P Marchetti

**Affiliations:** 1 Orthopedics, University of New England, Biddeford, USA; 2 Sports Medicine, Eastern Connecticut Health Network, Manchester, USA

**Keywords:** rotator cuff pathology, fall injury, cervical pain, brachial plexopathy, orthopedic sports medicine, clinical examination, reversible cause of muscle weakness, parsonage turner syndrome

## Abstract

Parsonage-Turner syndrome (PTS), also referred to as idiopathic brachial plexopathy or neuralgic amyotrophy, is a rare disorder that classically presents with abrupt, patchy, unilateral shoulder pain followed by varying degrees of weakness and atrophy of the upper extremity musculature. PTS is a serious diagnosis that can result in irreversible atrophy with delayed diagnosis and treatment. Since there currently is no gold standard for diagnosis and the syndrome can present as many other possible pathologies, careful clinical examination and thorough review of relevant imaging and diagnostic studies are critical for proper diagnosis. Here, we present a case of PTS diagnosed in a 67-year-old male with extensive overlapping cervical and rotator cuff pathology following an apparent orthostatic episode with no known mechanism of injury. This case report not only adds to the literature regarding the importance of close examination and plausible etiologies of PTS but also emphasizes close collaboration among specialties to avoid misdiagnosis.

## Introduction

Parsonage-Turner syndrome (PTS), also referred to as idiopathic brachial plexopathy or neuralgic amyotrophy, is a rare disorder that classically presents with abrupt, patchy, unilateral shoulder pain followed by varying degrees of weakness and atrophy of the upper extremity musculature [1-4]. The overall incidence of PTS has been cited at about 1.64 per 100,000 individuals in the general population [5].

Although the etiology of PTS is primarily unknown, there are numerous etiologies cited in the literature, including autoimmune diseases, hereditary factors, post-routine vaccinations, trauma from manipulation of tissues, post-viral infections, and patient positioning following postsurgical procedures [3,6-7]. The pathophysiology of PTS continues to remain elusive but may involve an interaction between genetic predisposition, mechanical vulnerability, and autoimmune triggers [2]. The initial pain of PTS is described as acute and unusually severe, with relentless pain in the neck, shoulder, or arm regions seen in 96% of patients [4]. Discomfort can last for several weeks, with one study reporting more than 10% of patients having initial pain lasting more than 60 days and more than 75% of patients who experienced two additional phases of position-dependent neuropathic pain lasting several months. This same study documented pain primarily at night in 60% of patients [3]. PTS is typically a self-resolving process, with 80-90% recovery of muscle strength within 2-3 years, but residual paresis and exercise intolerance can happen in up to 70% of patients [6,8]. Sensory deficits are less prevalent than motor symptoms but are still often present, with Cwik et al. reporting deficits in 66% of patients [9]. In one of the largest natural history studies of PTS, Tsairis et al. found that 89% of patients had fully functional recovery at three years [10-11].

Traditionally, the acute neuropathic pain of PTS is managed with long-acting opioid and nonsteroidal anti-inflammatory drugs (NSAID), such as diclofenac, and the musculoskeletal pain caused by resultant abnormal muscle compensation is primarily addressed by physical therapy to restore normal biomechanical motion. Oral corticosteroids can also help alleviate pain and accelerate recovery [3-4]. Recently, there has been some degree of evidence suggesting benefits from the administration of immunoglobulins, with some patients responding positively, but there are little data, and more rigorous research is required to determine their ultimate efficacy [12]. Although not preferred, if conservative treatment fails, patients may need surgical intervention. Decompression and microneurolysis of the affected nerves, specifically the long thoracic nerve, have demonstrated improvement in pain reduction and stability of the upper extremity [2].

PTS is a particularly difficult syndrome to diagnose because it is primarily a diagnosis of exclusion, and tests, such as routine blood examinations, CT, MRI, or electromyography (EMG), are used to rule out other disorders with overlapping symptoms [2,8]. Among possible differential diagnoses include rotator cuff pathology, cervical radiculopathy, post-herpetic neuralgia, amyotrophic lateral sclerosis (ALS), osteoarthritis of the cervical spine, and adhesive capsulitis [4,11,13].

A thorough review of the literature through the Pubmed and Cochrane databases yielded a small number of case reports of patients developing PTS following a traumatic fall. However, to the best of our knowledge, no case report exists of PTS emerging in a patient without a clear mechanism of injury with concomitant cervical and rotator cuff pathology obscuring the diagnosis. Here, we present a case of a patient who presented to the office with a sudden loss of function in his left arm following a fall and who was diagnosed with PTS after extensive workup and specialist consultation.

## Case presentation

The patient is a 67-year-old male with a past medical history significant for cervical C6-C7 discectomy and cervical fusion in 1993. He reported about 10 days prior to the presentation that he was sitting at his home when he stood up rather quickly, suffered an apparent orthostatic episode, and fell directly onto his face. There was no information from the patient about what may have caused or led to the fall, as the patient does not recall any details other than standing up quickly. There was no loss of consciousness reported by the patient. He had full use of his left arm prior to the fall, and according to the patient, the loss of function occurred seconds to minutes after this fall. The patient described not being able to lift his arm and having difficulty picking up objects. All symptoms were completely new since the fall. He had not seen any other providers prior to this and had no recent imaging performed. A review of systems was positive for syncope without loss of consciousness, minimal to no pain in the left upper extremity, neck pain, and stiffness, as well as weakness, numbness, and tingling in the left upper extremity. He denied ataxia, confusion, or dizziness pre- or post-episode.

On physical exam, he is obese with a BMI of 32 but had an otherwise unremarkable physical appearance. He displayed significant motor and sensory deficiencies in the left arm. His left shoulder demonstrated decreased motion in all planes but was notably worse in flexion and abduction with significant loss of strength in the rotator cuff muscles and deltoid. No swelling or tenderness was appreciated. The right shoulder was normal in strength and range of motion. The left elbow also demonstrated decreased range of motion in both flexion and extension. Examination of his left hand revealed a significantly decreased range of motion in the wrist with decreased strength in finger abduction, thumb/finger opposition, and wrist extension. He also displayed decreased sensation in the ulnar and median nerve distributions. There was notable weakness and atrophy in the left biceps brachii muscle. Visually, the patient did not have significant muscle mass, and therefore rotator cuff muscles were more difficult to determine whether there was atrophy present.

His reflexes were also markedly diminished bilaterally: triceps reflexes were 1+ on the right and 0 on the left, biceps reflexes were 1+ on the right and 0 on the left, and brachioradialis reflexes were 1+ bilaterally. The cervical spine had decreased range of motion and notable crepitus with movement but was without swelling, deformity, rigidity, or tenderness. Due to the patient's presenting symptoms and history of cervical fusion, the initial concern was a new onset disc herniation or damage of the previous fusion. Urgent neurosurgical consultation and imaging were requested because of the patient's significant weakness in the left shoulder, forearm, wrist, and hand.

**Figure 1:**
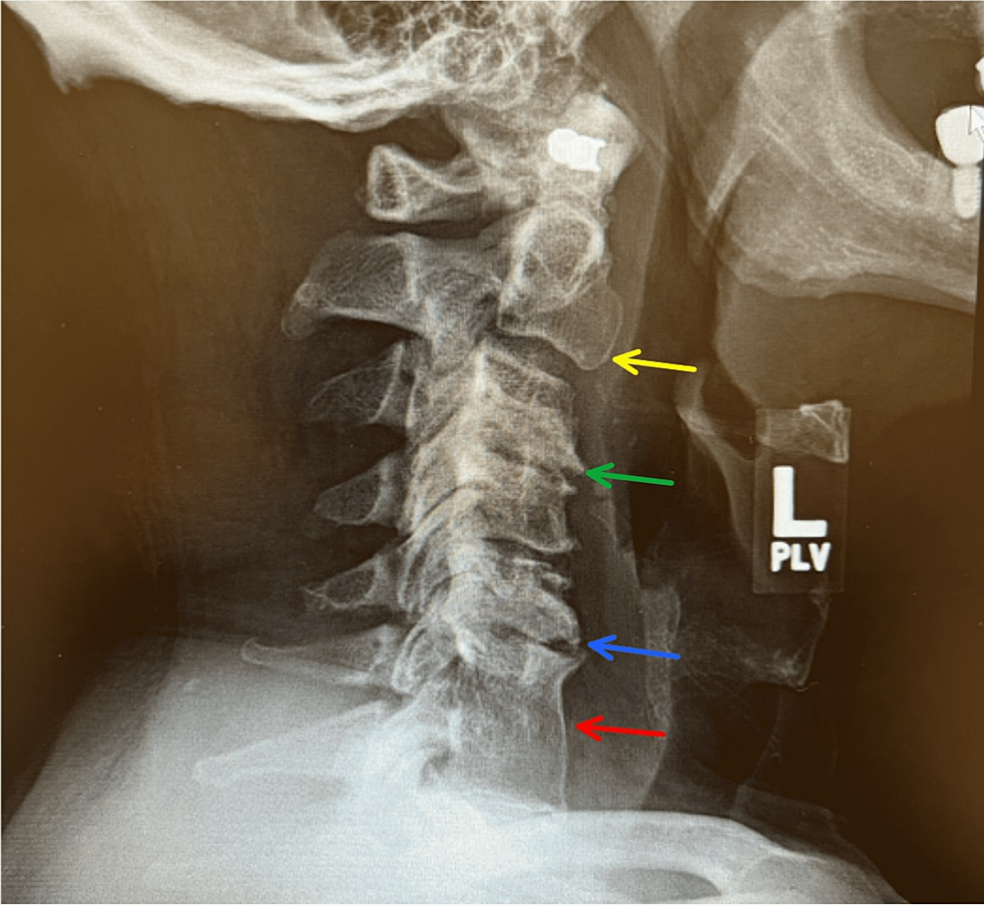
X-ray of the cervical spine Grade 1 C2 on C3 anterolisthesis (yellow arrow). Severe multilevel degenerative changes, particularly C3-C4 (green arrow) and C5-C6 (blue arrow). Narrowing of the disc spaces, anterior dorsal endplate with sclerotic degenerative changes. The old C6-C7 discectomy and fusion (red arrow).

**Figure 2:**
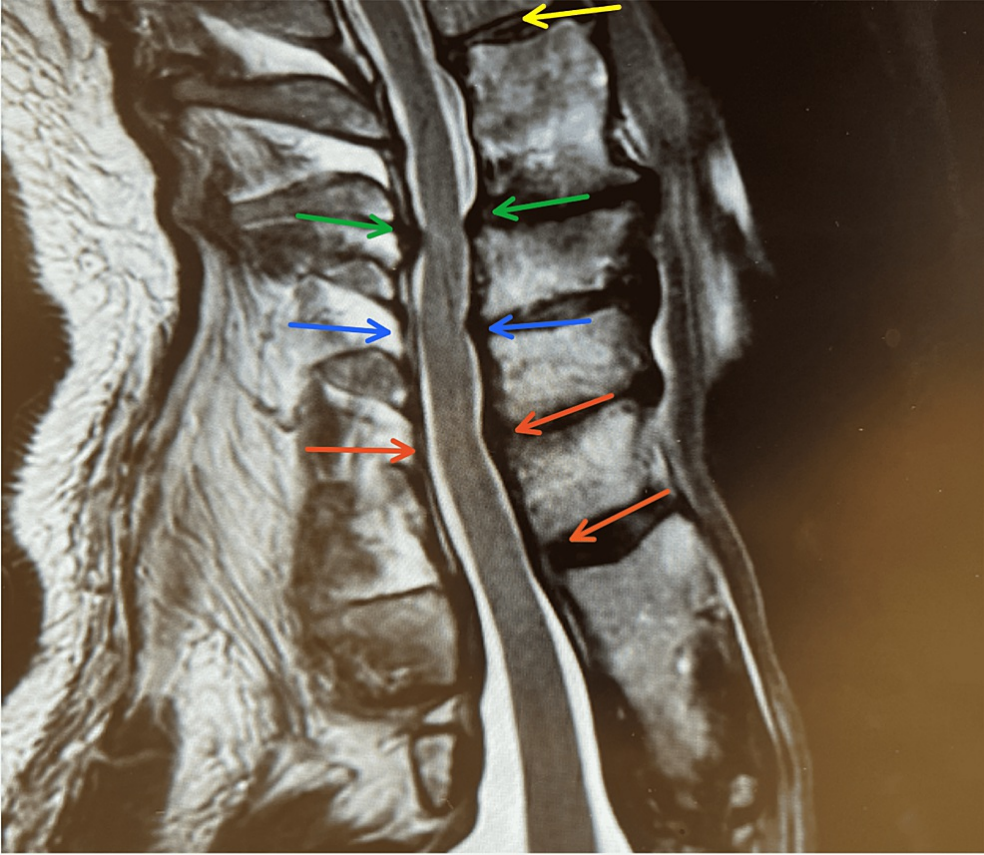
MRI of the cervical spine C2-C3: Small central disc protrusion and bilateral facet arthrosis causing mild central canal stenosis without neuroforaminal narrowing (yellow arrow). C3-C4: Bilateral facet arthrosis and uncovertebral joint osteophytes causing mild to moderate bilateral neuroforaminal narrowing without central canal stenosis. Bilateral foraminal nerve root encroachment (green arrows). C4-C5: Central disc protrusion causing moderate central canal stenosis. There were bilateral uncovertebral joint osteophytes and facet arthritis causing severe right and moderate left neuroforaminal narrowing with right foraminal C5 nerve root impingement and left foraminal C5 nerve root encroachment (blue arrows). C5-C6: Posterior disc osteophyte complex causing moderate to severe central canal stenosis. There are bilateral uncovertebral joint osteophytes and facet arthrosis resulting in severe bilateral neuroforaminal narrowing with bilateral foraminal nerve root impingement (red arrows). C6-C7: No central canal stenosis or neuroforaminal narrowing (orange arrow).

X-ray of the C-spine revealed grade 1 C2 on C3 anterolisthesis, severe multilevel degenerative changes (particularly C3-C4 and C5-C6), narrowing of the disc spaces, anterior dorsal endplate with sclerotic degenerative changes, and the old C6-C7 discectomy and fusion (Figure 1). MRI of the cervical spine displayed multilevel cervical spondylosis, bilateral foraminal C4 nerve root encroachment, right foraminal C5 nerve root impingement, left foraminal C5 nerve root encroachment, and bilateral foraminal C6 nerve root impingement. Nerve roots C7-T1 showed bilateral facet arthrosis and bilateral foraminal disc protrusions, causing mild bilateral neuroforaminal narrowing without central canal stenosis or impingement (Figure 2). MRI of the shoulder demonstrated mild supraspinatus, infraspinatus, and subscapularis tendinosis, as well as moderate bicipital tendinosis with a concern for tearing of the posterior-superior and anterior-inferior labrum. 

There was an initial bias from the neurosurgical consultation that his injury was cervical in nature. Due to the discrepancy between the patient's imaging studies and his clinical examination, an EMG was eventually performed, which revealed bilateral brachial plexopathies consistent with Parsonage-Turner syndrome. Needle EMG of the left supraspinatus, infraspinatus, deltoid, biceps, triceps extensor digitorum communis, and the right biceps showed 1+ to 3+ spontaneous activity with decreased recruitment. Nerve conduction studies showed prolonged peak latency in the right median sensory response, while the left showed diminished amplitude. The right ulnar sensory response showed diminished amplitude, while the left was absent. The left radial sensory response displayed diminished amplitude while the right was normal. Overall, the plexopathy was more significant on the left, affecting the superior, posterior, and inferior trunks while affecting the superior trunk on the right. There was additional evidence of superimposed cervical radiculopathy from the C5-C7 levels, as well as mild to moderate median neuropathy at the right wrist. The patient did not require any urgent intervention and is now improving in physical therapy.

## Discussion

Though PTS is uncommon, it is important in orthopedic practice because it requires differentiation from other musculoskeletal pathologies, and ultimately, early diagnosis of PTS will modify the prognosis of the patient [13]. A sample of differential diagnoses can be seen in Table 1 below, adapted from Franz et al. and van Alfen [2,8]. Initial workup for any patient presenting with acute upper extremity pain or weakness should include imaging studies and diagnostics. Presently, the EMG is the only modality that can definitively support a diagnosis of PTS [14]. Although diagnostics are important for supporting the PTS diagnosis, a complete physical exam with extensive manual muscle testing is essential before performing any specific studies [11]. A targeted clinical examination focusing on the serratus anterior, biceps brachii, and deltoid muscles in addition to the rotator cuff should be performed [3,7], as Dumitru et al. reported that 50% of patients with PTS displayed motor abnormalities in this trio of muscles [1].

**Table 1:** Differential diagnoses of PTS Adapted from Franz et al. and van Alfen [2,8].

Orthopedic/neurologic/other disorders	Distinguishing features
Parsonage-Turner syndrome	Acute onset, severe neuropathic pain, irregular nerve distribution, acute self-resolving muscle weakness.
Rotator cuff pathology	Subacute or insidious onset, pain intensity varies, worsens with motion, weakness, progressive.
Adhesive capsulitis	Subacute onset, pain, "frozen shoulder", limited range of motion (active and passive), progressive.
Subacrombial bursitis	Subacute onset, pain along front and side, nighttime pain, painful arc of motion, fluctuating course.
Calcific tendinitis	Subacute onset, pain, worsens with elevation, sometimes stiffness, self-limiting.
Osteoarthritis	Insidious onset, pain, worsens with motion, stiffness, secondary weakness, slowly progressive.
Facioscapulohumeral dystrophy	Onset during adolescence, facial weakness, weakening biceps/triceps/deltoids, hearing loss, painless, progressive.
Cervical spondylosis with referred brachialagia	Often posture or activity-dependent, no neurological deficits, fluctuating course.
(Primary) tumors of the scapula	Non-acute onset, scapular winging without weakness and no neurological symptoms.
Cervical radiculopathy, degenerative	Insidious onset, slowly progressive or fluctuating course.
Cervical radiculopathy, disc rupture	Acute onset, pain varies with posture: pain, sensory, and motor symptoms occur in the same dermatome.
Mononeuritis multiplex/vasculitis	Symptoms also occur in the legs or distal arm, subacute onset, progressive.
Multifocal motor neuropathy	Painless, no sensory symptoms, distal predominance, progressive.
Asian tick-borne encephalitis (poliomyelitic)	Following viral prodrome, severe headache and back pain, flaccid shoulder girdle paralysis.
Focal motor neuron disease	Insidious onset, no sensory symptoms, painless, progressive.
Entrapment neuropathies	Subacute onset, mild to moderate pain, prominent sensory symptoms.
Complex regional pain syndrome	Subacute onset, vasomotor features predominate, diffuse pain and weakness, progressive.
Lyme disease	Subacute onset, swelling, pain, rash, fever, fatigue, fluctuating course.

Medial winging of the scapula is the most frequently reported clinical sign of PTS, occurring in about two-thirds of patients [8], but it is also important here to point out that any nerve and, therefore, muscle innervated by the brachial plexus can be involved. The serratus anterior was not assessed on our physical exam for this patient, and the long thoracic nerve was not included in EMG studies. When testing individual muscles, it is essential to put the patient in the proper position, which will prevent the detection of false weakness in the rotator cuff or deltoid that could result in a misdiagnosis by identifying a false lesion within a peripheral nerve [7]. Since the phrenic nerve is also often involved, observing the paradoxical elevation of the diaphragm is another clinical clue. About one-third of PTS attacks also have bilateral involvement and are asymmetrical in severity; therefore, the examiner must pay close attention to the muscles that the patient doesn't mention to ensure the examination is as detailed as possible before considering a final diagnosis, as was the case in this patient [4].

After the clinical exam, an EMG can be used to display common nerve distributions associated with PTS, support clinical findings, and further rule out other pathologies. The suprascapular nerve, long thoracic nerve, axillary nerve, and phrenic nerve have been found to be the most heavily implicated nerves affected in PTS. Less reported nerves include the anterior interosseous, musculocutaneous, and spinal accessory nerves, with the radial, ulnar, and median nerves being the least involved [3,10-11]. The EMG will be able to display an irregular pattern of nerve distribution classic to PTS and will characteristically display numerous denervation signs in a neurogenic pattern. In PTS, velocity conduction will also remain normal, excluding demyelination disorders [13].

Overall, the EMG and nerve conduction studies supported the clinical findings found in our patient. On physical exam, the patient's left arm strength was noted to be weakest in flexion and abduction, which coincided with deficiencies found in the left supraspinatus, infraspinatus, and biceps brachii muscles on the EMG. On the left side, the plexopathy affected all three trunks to a degree, which resulted in the muscular deficiencies observed on the exam, though specific nerves within the trunks were not delineated. His sensory exam also yielded deficiencies in the median and ulnar nerve distributions, which were also found in nerve conduction studies. A radial nerve sensory distribution was not appreciated on the exam but was also found in the conduction study. His weakness in wrist and elbow extension on the exam, as well as decreased recruitment in the left triceps and extensor digitorum communis found on EMG, suggests the nerve and possibly its branches were indeed involved.

The MRI can also sometimes present subtle clues to suggest PTS. For example, a patient with PTS can display a high signal intensity involving one or more muscles innervated by the brachial plexus depicted on a T2 weighted image, which reflects increased capillary blood volume in the denervated muscle. T1 weighted imaging can also show focal fatty atrophy in 30% of cases [2,15], though MRI is generally not used in diagnosis as only 6.3% of patients have an altered study, and these signs are not specific to PTS [13]. In this case, it was imperative that his MRI findings of rotator cuff tendinosis and possible tear be interpreted closely to make sure these findings correlated with all aspects of the physical examination, which again was not the case for the patient. In addition, the patient did not experience any motion-dependent pain, which would be expected in a rotator cuff pathology. 

The patient also had coexistent severe narrowing and nerve impingements at multiple levels of the cervical spine. In this case, ruling out cervical radiculopathy (CR) was much more difficult primarily because CR is also a diagnosis of exclusion with similarly presenting upper extremity symptoms. Based on our patient's prior cervical fusion and longstanding changes in his cervical spine, this diagnosis initially made the most sense. Neurosurgery initially heavily favored this diagnosis, but with PTS, it is extremely important not to anchor to a particular diagnosis if the clinical examination does not correlate with imaging findings. If our patient's lesions were purely radicular, his sensory symptoms and paresis within affected muscles would more than likely be present in a specific dermatomal pattern traced back to a specific nerve root. This patient displayed decreased sensation in the median and ulnar nerve distributions, which does not correlate with the expected dermatomal pattern if this injury really was purely cervical in origin.

For example, the patient also demonstrated weakness with left digit abduction. This function is carried out by the interosseous muscles, which in turn are innervated by the ulnar nerve. The ulnar nerve is primarily derived from the C7/C8-T1 distribution, which does not support a diagnosis of radiculopathy because although MRI of his cervical spine displayed C7-T1 bilateral neuroforaminal narrowing and some degree of superimposed radiculopathy in C7, there was no definitive evidence of nerve impingement in these nerve roots. 

Many individuals with positive cervical imaging findings may be completely asymptomatic. The presence of cervical disc herniation, spondylosis, or osteophytic spurring of the intervertebral foramen does not equate to a CR diagnosis with certainty. Another distinguishing factor is that the onset of CR is normally insidious [12], whereas our patient had an acute presentation following his fall. Interestingly, Feinberg et al. suggest a physician consider CR and PTS in the differential to administer a cervical epidural to distinguish PTS from radicular pain [11]. In this case, his acute weakness in the extremity can most likely be attributed to PTS, but with his diminished reflexes and observable atrophy, this could also be attributed to his superimposed acute on chronic radiculopathy observed in the patient's MRI. One can postulate that the fall exacerbated his cervical pathology, which subsequently triggered the PTS, as trauma from manipulation of tissues has been cited as a plausible etiology of PTS [6]. Again, the patient had no history of these symptoms; therefore, this theory cannot be proven, which makes this case especially unique. Given the patient's minimal pain, this could also mean that his PTS was not caused by the fall but, in fact, was something developing over time.

## Conclusions

PTS is a disease that spans multiple specialties, including primary care, sports medicine, orthopedic surgery, and neurology. Even with close examination, close collaboration among specialties is particularly important because if each provider isn't on the same page in terms of the origin of the patient's presenting symptoms, this can result in delayed diagnosis and subsequent muscle atrophy.

In conclusion, PTS is a serious diagnosis that can result in irreversible atrophy with delayed diagnosis and treatment. Since there is currently no gold standard for diagnosis and the syndrome can present as many other possible pathologies, careful clinical examination and thorough review of relevant imaging and diagnostics is critical for proper diagnosis. Although there are patterns one can look for that will support a diagnosis, the irregularity of nerve distribution of PTS makes it even more difficult to diagnose, making close collaboration among specialties vital for preventing delayed diagnosis. 
